# A national framework for managing dual-use research of concern: integrating biosecurity, public health, and research governance

**DOI:** 10.3389/fbioe.2026.1924238

**Published:** 2026-08-31

**Authors:** Ruihan Zhang

**Affiliations:** School of Population Health, Faculty of Medicine and Health, UNSW Sydney, Sydney, NSW, Australia

**Keywords:** biorisk management, biosafety, biosecurity, cyberbiosecurity, dual-use research of concern, health security, research governance

## Abstract

Dual-use research of concern (DURC)—legitimate life-science research that could be misapplied to cause significant harm—has become an increasingly urgent policy challenge. Advances in synthetic biology, gene editing, and artificial intelligence have expanded both the pace and diffusion of high-risk biological work, while governance systems remain fragmented across national boundaries. Based on a structured narrative policy review updated through 28 July 2026, this article proposes a comprehensive national DURC risk-management program that bridges biosecurity, public health, research governance, and emergency management. Drawing on the World Health Organization’s 2022 Global Guidance Framework for the Responsible Use of the Life Sciences, the ISO 35001:2019 biorisk management standard, and national policy precedents—including the design of the 2024 U.S. Government Policy for Oversight of Dual Use Research of Concern and Pathogens with Enhanced Pandemic Potential (DURC-PEPP Policy), whose planned implementation was superseded by Executive Order 14,292 while a revised or replacement federal policy remained under development at the final search date—the article maps five overlapping threat pathways relevant to DURC governance, analyzes their multi-layered consequences, and identifies existing tools and stakeholder interfaces that can be adapted. The core of the framework comprises eleven integrated recommendations across prevention and response domains: establishing a national DURC governance structure, strengthening institutional oversight and ethics review, implementing laboratory biorisk management systems, addressing cyberbiosecurity, mitigating insider threats, promoting responsible communication safeguards, enhancing early detection and surveillance, developing DURC-specific emergency response plans, protecting first responders and healthcare workers, institutionalizing risk communication and public engagement, and embedding continuous learning and international collaboration. The program emphasizes the protection of frontline workers, the integration of occupational health into biosecurity planning, and the institutionalization of intersectoral coordination mechanisms. Achieving these goals requires sustained collaboration across health, security, research, digital, and community sectors, but the benefits extend beyond DURC, enhancing national preparedness for all biological threats.

## 1 Introduction

### 1.1 Defining the problem

Dual-use research of concern (DURC) describes legitimate life-science research that is reasonably anticipated to generate knowledge, products, or technologies that could be misapplied to cause significant harm to humans, animals, plants, or the environment (Ienca and Vayena, 2018). The tension at the heart of DURC governance is not new, but its urgency has intensified dramatically. Recent analyses highlight that advances in synthetic biology, gene editing, and artificial intelligence (AI)-enabled design tools are lowering technical barriers faster than regulatory systems can adapt (Cummings et al., 2021; Hoffmann et al., 2023). DURC can therefore increase the risk and potential consequences of high-consequence biological incidents arising from laboratory accidents, insider misuse, cyber-enabled compromise of laboratory systems, or deliberate misuse of biological agents.

In parallel, emerging governance gaps have been documented, including fragmented national rules, uneven institutional oversight, and limited integration of security perspectives into research ethics and funder review processes (Gillum, 2025a; Salloch, 2018). These gaps are not merely academic concerns: the COVID-19 pandemic demonstrated how quickly biological events can overwhelm health systems and exposed weaknesses in biosafety, biosecurity, and surge capacity (Rutjes et al., 2023). The pandemic also intensified debates about the origins of high-consequence pathogens, bringing DURC governance from a niche policy concern into mainstream public discourse (Gillum, 2025a; World Health Organization, 2022).

These converging pressures motivate the development of a coherent national DURC risk-management program that links biosecurity, public health, research governance, and emergency management into a single integrated framework—an approach that the World Health Organization (WHO) has explicitly called for in its 2022 Global Guidance Framework (World Health Organization, 2022).

Illustrative examples identified in this review include governance approaches or implementation initiatives in the United States, Denmark, and Uganda (United States Office of Science and Technology Policy, 2024; Onkenhout, 2024; World Health Organization, 2023). These approaches vary substantially in scope, legal authority, and implementation capacity. The governance gap is therefore not a complete absence of national action, but rather its fragmentation and uneven global coverage, particularly in low- and middle-income settings.

### 1.2 Motivation for developing a national DURC program

Several developments strengthen the case for a dedicated national program. First, technological acceleration in gene editing, low-cost DNA synthesis, and laboratory automation has expanded the scale, speed, and diffusion of high-risk biological work, amplifying both potential benefits and risks (Ienca and Vayena, 2018; Hoffmann et al., 2023). Benchtop DNA synthesizers that once cost hundreds of thousands of dollars are becoming accessible to smaller laboratories and even non-institutional actors, fundamentally reshaping the threat landscape (Hoffmann et al., 2023; Nuclear Threat Initiative, 2025).

Second, growing evidence of laboratory incidents and escapes is worrying. A scoping review published in *The Lancet Microbe* documented dozens of laboratory-acquired infections and pathogen escapes worldwide between 2000 and 2021, including incidents involving SARS-CoV, Ebola, and highly pathogenic avian influenza (Blacksell et al., 2024). These are not theoretical risks; they represent documented system failures.

Third, the COVID-19 pandemic exposed substantial biosafety, biosecurity, laboratory-operational, and surge-capacity challenges across public-health systems (Rutjes et al., 2023). Comparable weaknesses could be especially consequential in an event involving an enhanced or deliberately released biological agent.

Fourth, renewed global governance efforts are raising expectations for stronger national systems. The WHO’s 2022 Global Guidance Framework for the Responsible Use of the Life Sciences calls on states to adopt integrated, life-cycle approaches to mitigating biological risks and governing DURC (World Health Organization, 2022). In parallel, the Ninth Review Conference of the Biological Weapons Convention (BWC) established a Working Group to strengthen the Convention, including through the consideration of measures for national implementation (United Nations, 2022). A 2025 policy institute report from the Nuclear Threat Initiative further documented how the BWC’s pathogen-centric framework is being challenged by emerging technologies, including AI-enabled synthetic biology (Nuclear Threat Initiative, 2025).

Finally, the economic dimension cannot be ignored. The World Bank has described the COVID-19 pandemic as the largest global economic crisis in more than a century, with disproportionate impacts on emerging economies and disadvantaged populations (World Bank, 2022). The scale of pandemic-related economic disruption underscores the potential value of sustained investment in biological threat prevention, including proportionate DURC governance.

### 1.3 Novel contribution and program aims

Rather than proposing an entirely new regulatory instrument, this review integrates existing DURC and biorisk governance tools into a national prevention–response architecture. Its proposed contribution lies in three areas. First, integration: the World Health Organization (WHO) Global Guidance Framework (2022), ISO 35001, and the U.S. DURC-PEPP Policy each address important dimensions of DURC governance (World Health Organization, 2022; United States Office of Science and Technology Policy, 2024; International Organization for Standardization, 2019). However, no single instrument identified in this review operationalizes all of these domains within one national prevention–response architecture encompassing DURC oversight, biosafety, biosecurity, cyberbiosecurity, public-health preparedness, occupational health, and emergency management, with designated interfaces among these domains. Second, implementation sequencing: the three-tier, dependency-based phasing proposed in Table 1 provides a practical pathway for countries to prioritize capabilities according to institutional maturity and resource availability. Third, the framework explicitly elevates occupational health, frontline worker protection, and safeguards against unintended consequences—including impacts on scientific freedom, equity for low- and middle-income countries (LMICs), and the potential for discriminatory insider-threat screening—from peripheral or implicit concerns to core framework components.

**TABLE 1:** Implementation priority matrix for the national DURC program.

Tier	Timing	Components	Rationale
Tier 1 (Immediate)	0–12 months	National governance framework (3.1.1); Institutional oversight with DURC screening (3.1.2); Early detection and surveillance (3.2.1)	No program can function without legal authority, institutional mandate, and situational awareness. Evidence profile: predominantly consensus-based; see Table 2
Tier 2 (Intermediate)	12–36 months	Laboratory biorisk management (ISO 35001) (3.1.3); Insider threat mitigation (3.1.5); Emergency response plans (3.2.2); Worker protection (3.2.3); Risk communication (3.2.4)	Requires Tier 1 governance to designate responsible entities and allocate resources. Evidence profile: mixed across recommendations; see Table 2
Tier 3 (Advanced)	36+ months	Cyberbiosecurity assessment (3.1.4); Responsible communication safeguards (3.1.6); Continuous learning and international collaboration (3.2.5)	Depends on mature Tier 1–2 systems and sustained political commitment; may exceed near-term capacity in lower-resource settings. Evidence profile: primarily author synthesis, with consensus support for continuous learning; see Table 2

Timelines are illustrative and indicate recommended sequencing and expected maturation rather than fixed completion deadlines. Foundational planning for emergency response, worker protection, risk communication, and continuous learning should begin during Tier one even where full implementation is assigned to a later tier. Implementation speed should be adapted to national legal processes and institutional capacity.

The proposed national program aims to:

1. Reduce the likelihood that DURC conducted within, or affecting, the country will contribute to a high-consequence biological incident or outbreak;
2. Limit the health impacts of any DURC-related biological incident or outbreak through rapid detection and coordinated response;
3. Protect first responders and frontline healthcare workers, laboratory workers, and public-health staff through robust occupational health and safety, biosafety, and biosecurity systems;
4. Embed ethical, legal, and governance safeguards into the life-sciences research cycle, from funding decisions to publication; and
5. Strengthen intersectoral and international collaboration, aligning health, security, research, and digital-security actors around shared biological risk goals (World Health Organization, 2022; Millett, 2023).

### 1.4 Scope of this review

This Policy and Practice Review defines key DURC-related threats and their potential consequences, maps existing tools and frameworks that can support a national program, identifies stakeholders and intersectoral considerations, proposes eleven prevention and response components, and discusses limitations and future research directions. Full implementation of the proposed framework assumes a national context with an operational life-science research sector, publicly funded research institutions, and access to at least biosafety level 3 (BSL-3) containment or equivalent high-consequence diagnostic and reference laboratory capacity. Where these preconditions are absent or embryonic, a tiered rollout is envisaged: countries could begin with foundational governance architecture and surveillance capability, then phase in laboratory biorisk management systems, reserving resource-intensive elements such as cyberbiosecurity audits and formal insider-threat screening for later stages as institutional capacity matures. Domestic private-sector laboratories operating within the national jurisdiction are included as stakeholders in the proposed framework; however, the governance of multinational private-sector research spanning multiple regulatory jurisdictions falls outside the present scope. The framework is therefore intended as a transferable national architecture rather than a country-specific regulatory model; its components and sequencing should be adapted to domestic legal authority, institutional maturity, financing, and research capacity.

### 1.5 Approach

This study used a structured narrative policy review approach. Relevant literature was initially identified during the development of the original master’s coursework using electronic resources available through the UNSW Library. Complete records of the specific databases, search dates, and numbers of records identified during this initial stage were not retained. The literature was subsequently updated through 28 July 2026 using PubMed, supplementary Google Scholar academic searches, targeted searches of official institutional websites, and citation tracking from eligible sources. The updated search used combinations of the terms “dual-use research of concern,” “biosafety,” “biosecurity,” “cyberbiosecurity,” “insider threat,” “biorisk management,” and “health security governance.”

Sources were included if they addressed DURC governance, PEPP oversight, laboratory biorisk management, cyberbiosecurity, insider-threat mitigation, public-health preparedness, or related national and international policy instruments. Sources focusing solely on technical laboratory procedures without governance dimensions were excluded. Potentially relevant sources were initially screened using titles, abstracts, executive summaries, or document descriptions, as available, followed by full-text review against the inclusion and exclusion criteria. The final synthesis comprised 37 cited sources. Because complete logs of the initial coursework-based search were not retained, the approximate numbers of records initially identified, deduplicated, screened, and excluded cannot be reconstructed.

The evidence base comprises four source groups. The first group includes international legal and normative instruments governing biorisk: the WHO Global Guidance Framework for the Responsible Use of the Life Sciences (World Health Organization, 2022), the Final Document of the Ninth Review Conference of the Biological Weapons Convention (United Nations, 2022), ISO 35001:2019 (International Organization for Standardization, 2019), the Biological Weapons Convention (United Nations, 1972), the International Health Regulations (2005) (World Health Organization, 2005), and the Joint External Evaluation Tool (World Health Organization, 2022). The second group includes national and regional policy and regulatory documents, notably the 2024 U.S. DURC-PEPP Policy (United States Office of Science and Technology Policy, 2024), the EU Dual-Use Regulation 2021/821 (European Union, 2021), Executive Order 14,292 (Executive Office of the President, 2025), and subsequent NIH guidance concerning the policy’s revision or replacement (National Institutes of Health, 2025). The third group comprises peer-reviewed journal articles and scholarly book chapters addressing DURC governance, biosafety, biosecurity, cyberbiosecurity, laboratory-acquired infections, and public-health emergency response (Ienca and Vayena, 2018; Cummings et al., 2021; Hoffmann et al., 2023; Gillum, 2025a; Salloch, 2018; Rutjes et al., 2023; Blacksell et al., 2024; Millett, 2023; Callihan et al., 2021; Crawford et al., 2023; Oladimeji et al., 2019; Morse, 2025; Sabra et al., 2024; Keogh-Brown et al., 2010; Evans, 2020; Murray et al., 2023; Jackson et al., 2006; Dietz, 2013; Mackey and Liang, 2012). The fourth group comprises grey literature, including doctoral and master’s theses, policy-institute reports, multilateral-organization publications, and program documents. These sources were retained when they provided primary policy, survey, or implementation evidence unavailable in peer-reviewed publications (Onkenhout, 2024; World Health Organization, 2023; Nuclear Threat Initiative, 2025; World Bank, 2022; Gillum, 2025b; Gronvall, 2013; Global Health Security Agenda, 2024; Pandemic Fund, 2024).

Policy documents were assessed according to their legal status, scope, oversight mechanisms, and enforceability. Evidence categories in Tables 2 and 3 were assigned through the author’s synthesis. “Consensus-based” indicates convergence across multiple authoritative frameworks or professional guidance documents; “mixed” indicates support from both empirical or observational studies and authoritative guidance; and “author synthesis” indicates recommendations extrapolated primarily from adjacent evidence, case studies, or emerging policy analysis. Peer-reviewed empirical and observational studies were appraised qualitatively according to study design, directness to the recommendation, methodological transparency, and consistency with other sources. Grey literature was assessed according to institutional provenance, methodological transparency, and relevance to policy implementation. No formal risk-of-bias tool was applied because the review combined heterogeneous policy instruments, legal texts, institutional reports, case studies, and scholarly literature. Given the narrative design, English-language restriction, incomplete records from the initial coursework-based search, and relatively small evidence base, selection bias cannot be excluded. Screening and source assessment were conducted by the author, and the absence of a complete initial search log limits the reproducibility of the review.

**TABLE 2:** Evidence basis for individual recommendations.

Recommendation	Tier	Evidence category	Principal basis
National DURC governance framework	1	Consensus-based	(World Health Organization, 2022; United Nations, 1972; United States Office of Science and Technology Policy, 2024)
Institutional oversight with DURC screening	1	Consensus-based	(Salloch, 2018; United States Office of Science and Technology Policy, 2024)
Early detection and surveillance integration	1	Consensus-based	(World Health Organization, 2005; World Health Organization, 2022; Cummings et al., 2021)
Laboratory biorisk management (ISO 35001)	2	Mixed (empirical + consensus-based)	(International Organization for Standardization, 2019; Callihan et al., 2021)
Insider threat mitigation	2	Author synthesis	(Gillum, 2025b; Murray et al., 2023)
DURC-specific emergency response plans	2	Mixed (empirical + consensus-based)	(World Health Organization, 2022; World Health Organization, 2005; Jackson et al., 2006)
First responder and worker protection	2	Mixed (empirical + consensus-based)	(World Health Organization, 2022; Blacksell et al., 2024; Rutjes et al., 2023)
Risk communication and public engagement	2	Consensus-based	(World Health Organization, 2022; Dietz, 2013)
Cyberbiosecurity assessment	3	Author synthesis	(Crawford et al., 2023; Nuclear Threat Initiative, 2025)
Responsible communication safeguards	3	Author synthesis	(Hoffmann et al., 2023; Evans, 2020; Gronvall, 2013)
Continuous learning and international collaboration	3	Consensus-based	(Morse, 2025; World Health Organization, 2022; Global Health Security Agenda, 2024)

Abbreviations: BWC, biological weapons convention; GHSA, global health security agenda; IBC, institutional biosafety committee; IHR, international health regulations; IRB, institutional review board; JEE, joint external evaluation; WHO, World Health Organization; DURC, dual-use research of concern; ISO, international organization for standardization; PEPP, pathogens with enhanced pandemic potential.

**TABLE 3:** Comparative analysis of major DURC and biorisk governance instruments.

Instrument	Legal status	Scope	DURC screening	Enforcement	LMIC applicability	Limitations
(World Health Organization, 2022)	Non-binding	Life sciences broadly	Yes (guidance)	Voluntary	High (designed for adaptation)	No enforcement mechanism
(International Organization for Standardization, 2019)	Voluntary standard	Laboratory biorisk	Implicit (risk-based)	External audit (voluntary)	Moderate (cost barrier)	Implementation and certification may require specialist expertise and additional cost
(United States Office of Science and Technology Policy, 2024)	Federal policy; planned implementation superseded by EO 14292 (21)	Federally funded research	Yes (explicit categories)	EO 14292-related funding restrictions; revised or replacement policy pending at final search (Executive Office of the President, 2025; National Institutes of Health, 2025)	Low (US-specific)	Gaps for privately funded work
(European Union, 2021)	Binding (EU law)	Export-controlled items	Partial (export focus)	National authorities	Low (EU-specific)	Limited R&D internal oversight
(United Nations, 1972)	Treaty	Biological weapons	No (weapons focus)	National implementation	Formally universal; implementation capacity varies	No verification mechanism
(World Health Organization, 2005)	Treaty	Public health emergencies	No (JEE assesses capacity) (World Health Organization, 2022)	Binding notification and reporting obligations; JEE participation is voluntary	Broad formal applicability; implementation capacity varies	Limited biosecurity specificity

LMIC, applicability reflects the author’s qualitative assessment of estimated cost, transferability, and institutional capacity requirements. These judgments are illustrative and should not be interpreted as empirically validated scores.

Abbreviations: BWC, biological weapons convention; DURC, dual-use research of concern; IHR, international health regulations; JEE, joint external evaluation; LMIC, low- and middle-income country; PEPP, pathogens with enhanced pandemic potential.

## 2 Landscape analysis: threats, existing instruments, and stakeholder considerations

### 2.1 Types of threats relating to DURC

Recent literature identifies five overlapping threat types relevant to DURC governance (Cummings et al., 2021; Gillum, 2025a). Understanding these categories is essential for designing proportionate, risk-calibrated controls.

#### 2.1.1 Laboratory accidents involving high-consequence biological agents

Work involving high-consequence pathogens, including research on pathogens with enhanced pandemic potential (PEPP), can result in accidental exposure or release when biosafety systems fail (Gillum, 2025a). The 1979 Sverdlovsk anthrax release—which killed at least 68 people—remains a severe documented example (Sabra et al., 2024), while laboratory-acquired SARS-CoV infections in Singapore and Taiwan in 2003–2004 demonstrate that even well-resourced laboratories remain vulnerable (Blacksell et al., 2024). Although these incidents did not necessarily involve modified pathogens or DURC, DURC- or PEPP-related research could amplify the consequences of comparable failures by involving biological agents with particularly serious public-health implications.

#### 2.1.2 Insider misuse

Staff with legitimate access to organisms, equipment, or sensitive information may act maliciously or under coercion. Insider threats remain among the most challenging risks in high-containment biological research, and mitigation requires integrated technical, procedural, and cultural measures—surveillance alone is insufficient (Gillum, 2025b). Recent foresight analyses further underscore how advances in synthetic biology and genome editing continue to amplify the accessibility and affordability of weaponizing pathogens such as *Bacillus anthracis* (Sabra et al., 2024).

#### 2.1.3 Cyberbiosecurity threats

As high-containment laboratories rely increasingly on networked instruments, automated systems, and digital sequence data, the attack surface for cyber-enabled biological harm expands. Cyber-attacks can manipulate experimental conditions, corrupt safety systems, or exfiltrate sensitive DURC data (Crawford et al., 2023). The convergence of cybersecurity and biosecurity—termed “cyberbiosecurity”—represents a relatively new and rapidly evolving governance concern that is not yet consistently integrated into DURC oversight frameworks (Nuclear Threat Initiative, 2025; Crawford et al., 2023).

#### 2.1.4 Unregulated or weakly governed research spaces

Small private laboratories, contract research organizations, and do-it-yourself biology communities may operate outside institutional oversight structures. While the DIY biology movement has generated valuable innovations and public engagement, its decentralized nature raises challenges for ensuring consistent biosafety and biosecurity standards (Cummings et al., 2021).

#### 2.1.5 Information and publication risks

Detailed methodological descriptions, genomic data, or vulnerability analyses in the open literature can lower barriers for hostile actors. Cummings’ review found that concerns about “information access” appeared in approximately one in ten articles discussing emerging biosecurity threats (Cummings et al., 2021). This tension between scientific openness and security—sometimes called the “dual-use dilemma of knowledge”—is particularly acute in fields such as synthetic virology and AI-enabled protein design (Nuclear Threat Initiative, 2025).

These five threat types differ substantially in their causal pathways and governance implications. Laboratory accidents primarily reflect biosafety failures and are best addressed through biorisk-management systems and physical containment. Insider misuse and bioterrorism involve deliberate human action and require personnel reliability programs, behavioral monitoring, and a security culture that encourages confidential reporting. Cyberbiosecurity threats exploit digital vulnerabilities and demand distinct technical controls alongside physical security measures. Unregulated research spaces and information hazards raise different challenges concerning oversight coverage and publication ethics. While all five threat types intersect with DURC governance—because DURC research amplifies the potential consequences of each—their prevention and mitigation strategies should be analytically distinguished before being integrated into a unified framework. Not every laboratory accident, cyber incident, insider event, or deliberate biological attack constitutes a DURC event. These pathways fall within the scope of the present framework only when the research, knowledge, technology, or biological materials involved meet the definition of DURC or involve research on pathogens with enhanced pandemic potential (PEPP).

### 2.2 Consequences of DURC-related biological incidents

A DURC-related biological incident or outbreak could have multi-layered impacts extending well beyond the clinical domain.

#### 2.2.1 Clinical consequences

Enhanced pathogens may transmit more efficiently, evade existing immunity, or resist available treatments, increasing morbidity and mortality relative to natural outbreaks. Even “ordinary” laboratory-acquired infections can be severe in vulnerable populations and produce downstream transmission; Blacksell et al. documented multiple cases where laboratory workers infected family members or community contacts (Blacksell et al., 2024).

#### 2.2.2 Health-system strain

COVID-19 demonstrated how surges of severe disease rapidly overwhelm hospitals, exhaust supplies, and delay routine care (Rutjes et al., 2023). DURC-related events may be even more challenging: early case definitions and diagnostics may be lacking, and the pathogen may have deliberately or inadvertently acquired traits that complicate clinical management.

#### 2.2.3 Occupational risks

First responders, emergency clinicians, public-health staff, and laboratory workers are often among the first exposed, yet their protection is frequently treated as secondary to patient care. The WHO framework emphasizes that worker protection is central to responsible life-science governance rather than an ancillary concern (World Health Organization, 2022).

#### 2.2.4 Socioeconomic and political impacts

A suspected or confirmed DURC-related outbreak could trigger stigma, protests, geopolitical tensions, and a profound loss of public trust in science and government. Past policy debates around high-risk research—particularly gain-of-function studies of pandemic potential—have already eroded trust in some communities, especially where transparency was limited (Gillum, 2025a). A DURC-related outbreak could also impose substantial economic costs, as illustrated by the broader economic consequences of COVID-19, SARS, and pandemic influenza (World Bank, 2022; Keogh-Brown et al., 2010; Mackey and Liang, 2012).

### 2.3 Means available to address the threat

**FIGURE 1:**
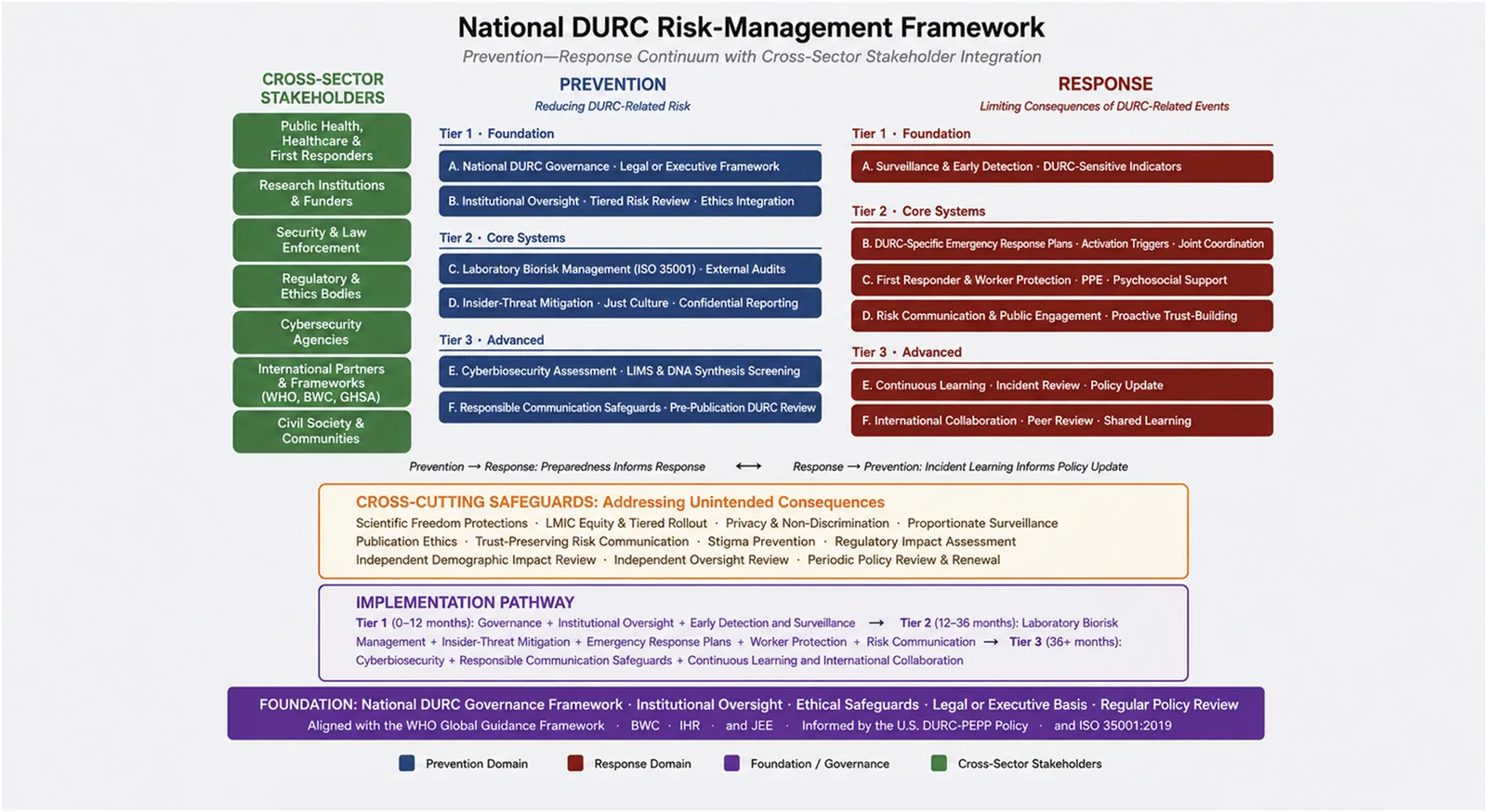
Architecture of the proposed national DURC risk-management framework. Prevention and response components are arranged by recommended implementation sequence. Cross-cutting safeguards address potential unintended consequences, while the implementation pathway supports context-specific, capacity-adapted adoption.

Countries are not starting from zero; several existing tools and frameworks provide a foundation on which a national program can build. Table 3 provides a comparative assessment of the major instruments, and Figure 1 illustrates the proposed framework architecture.

The WHO Global Guidance Framework (2022) sets out values, principles, and a comprehensive menu of tools for states to adapt (World Health Organization, 2022). It explicitly addresses the full research life cycle—from funding decisions through laboratory operations to publication—and emphasizes the importance of integrating biosafety, biosecurity, and bioethics considerations.

The ISO 35001:2019 standard provides a structured biorisk management framework for laboratories and related organizations (International Organization for Standardization, 2019). Evidence from the COVID-19 pandemic suggests that laboratories with structured risk-assessment and incident-learning systems were better able to adapt to new threats (Rutjes et al., 2023; Callihan et al., 2021).

At the regulatory level, the 2024 United States Government Policy for Oversight of DURC and Pathogens with Enhanced Pandemic Potential (DURC-PEPP) represented a detailed attempt to consolidate oversight of dual-use and enhanced-pathogen research. The policy set out categories of high-consequence research, additional institutional review requirements, and documented risk-mitigation plans. Its planned implementation was superseded following Executive Order 14,292 in May 2025 (Executive Office of the President, 2025). At the time of the final literature update on 28 July 2026, official NIH guidance continued to describe the 2024 policy as subject to revision or replacement and indicated that further guidance would be issued (National Institutes of Health, 2025). Although the policy was not implemented as originally planned, its design features remain an instructive policy precedent for countries developing national DURC governance mechanisms.

Beyond formal standards and regulations, scholarship on governance, ethics, and “safety by design” argues for integrating safety and security considerations upstream in project design, funding decisions, and technology platforms, rather than relying solely on end-stage controls (Hoffmann et al., 2023; Gillum, 2025a). This preventive philosophy—building security into the research process rather than bolting it on afterward—is a recurring theme in the recommendations that follow.

The European Union’s Dual-Use Regulation (2021/821) provides another governance model, focusing on export controls and research oversight for dual-use items. While its scope differs from a comprehensive DURC program, its mechanisms for inter-agency coordination and researcher engagement offer transferable lessons (European Union, 2021).

### 2.4 Stakeholders

Based on WHO guidance and recent analyses, a DURC program must engage multiple stakeholder groups (World Health Organization, 2022; Oladimeji et al., 2019).

- Public-health authorities: national and sub-national health departments, emergency operations centers, and public health laboratories;
- Healthcare providers and first responders: hospitals, ambulance services, primary-care clinics, and occupational-health services;
- Research institutions and funders: universities, public research institutes, private R&D laboratories, and governmental funding agencies;
- Security and law-enforcement agencies: police, intelligence services, border and customs authorities;
- Regulatory and ethics bodies: research ethics committees, institutional biosafety committees, and professional societies;
- Digital and cybersecurity actors: agencies responsible for protecting laboratory networks, cloud platforms, and sequence databases (Crawford et al., 2023);
- International partners: WHO, neighboring countries, Interpol, and global networks focused on biosecurity and DURC training;
- Civil society and community representatives: patient advocacy groups, science communication organizations, and affected communities who ensure transparency and public trust.

The inclusion of civil society is notable because DURC governance has historically been dominated by technical and security actors. Building public legitimacy for oversight decisions—particularly those that restrict research—requires meaningful engagement with communities who ultimately bear the risks and benefits of life-science advances (World Health Organization, 2022; Dietz, 2013).

### 2.5 Intersectoral considerations

Analyses of DURC governance consistently emphasize that no single sector can manage these risks alone (Ienca and Vayena, 2018; Millett, 2023). Key interfaces include.

- Health–security: sharing intelligence on suspicious events while respecting public-health confidentiality requirements;
- Health–science: linking surveillance data with research oversight so that unusual disease signals can be investigated for possible DURC links;
- Health–digital: aligning cyberbiosecurity protocols with infection-control and laboratory information systems;
- Health–labor: integrating occupational-health policies with biosecurity planning so that worker protection is embedded in, not separate from, biorisk management.

However, existing coordination is often episodic and personality-driven rather than institutionalized (Rutjes et al., 2023). The SARS (2003) and H1N1 (2009) outbreaks demonstrated how uncoordinated public health and economic responses can compound harm, prompting calls for formalized coordination mechanisms such as joint WHO–WTO committees to balance health protection with trade and economic interests (Mackey and Liang, 2012). Effective intersectoral collaboration requires regular joint exercises, shared incident-reporting platforms, designated liaison officers, and clear memoranda of understanding that survive personnel changes.

## 3 Recommendations for a national DURC risk-management program

The following eleven recommendations are organized across two domains: prevention (addressing the probability of a DURC-related event) and response (addressing the consequences should an event occur). The recommendations vary in evidentiary strength. As detailed in Tables 2 and 3, some draw on empirical or observational evidence, whereas others rely primarily on authoritative guidance, expert consensus, evidence from adjacent sectors, or author synthesis. They should therefore be interpreted as a proposed policy architecture for adaptation and evaluation rather than as a set of empirically validated interventions.

### 3.1 Prevention components

#### 3.1.1 Establish a national DURC governance framework

The foundation of any effective program is a national DURC and biological risk strategy that aligns with the WHO Global Guidance Framework (World Health Organization, 2022). This strategy should define DURC and pathogens with enhanced pandemic potential (PEPP) for national purposes; clarify the roles and responsibilities of ministries, regulators, funders, and research institutions; mandate that all publicly funded life-science research be screened for DURC potential at both funding and institutional levels; require high-consequence projects to undergo additional, independent review with documented risk-mitigation plans, drawing on design features of the 2024 U.S. DURC-PEPP Policy (United States Office of Science and Technology Policy, 2024); and commit to regular review as science and technologies evolve (Gillum, 2025a).

The governance framework should be established through legislation or executive order to ensure durability beyond any single administration. Where consistent with national legal arrangements, legislation or executive authority may provide greater institutional durability than informal administrative mechanisms; however, comparative evidence on the effects of different legal bases remains limited.

A critical design choice is whether to adopt a list-based approach (defining specific pathogens or experiment types subject to oversight) or a broader risk-based approach that considers the foreseeable consequences of research regardless of pathogen type. Millett argues persuasively that taxonomic lists alone are insufficient because they fail to capture novel or synthetic agents (Millett, 2023). The program should therefore combine a defined list of high-consequence agents with a risk-based assessment framework that can capture emerging threats.

#### 3.1.2 Strengthen institutional oversight and ethics review

Institutional biosafety committees and research ethics committees should be formally tasked with considering DURC issues as part of their review mandates. Salloch notes that these committees require specific expertise and guidance to address biosecurity consequences, not only traditional research ethics concerns such as participant protection (Salloch, 2018).

Practical measures include standardized DURC screening questions embedded in ethics and biosafety application forms; access to a national DURC advisory panel for complex or contentious cases; and mandatory training modules on dual-use dilemmas for committee members, principal investigators, and research students (World Health Organization, 2022; United States Office of Science and Technology Policy, 2024; Oladimeji et al., 2019). These training modules should include case studies—such as the 2011 H5N1 transmission controversy and the 2017 synthesis of horsepox virus—that illustrate the real-world ethical tensions researchers and reviewers face (Evans, 2020; Gronvall, 2013).

Institutional oversight should be proportionate to risk. An appropriately tiered system—where low-risk projects receive streamlined review while high-consequence work undergoes expanded assessment—may reduce oversight fatigue and resource bottlenecks while directing greater scrutiny to the most concerning research (World Health Organization, 2022; United States Office of Science and Technology Policy, 2024; Gillum, 2025b).

#### 3.1.3 Implement biological risk management systems in laboratories

All high-risk laboratories (BSL-3, BSL-4, major diagnostic and reference laboratories) should implement formal biological risk-management systems aligned with ISO 35001:2019 (International Organization for Standardization, 2019). Evidence from COVID-19 suggests that laboratories with structured risk-assessment, incident-reporting, and continuous-improvement systems were better able to identify and respond to emerging biosafety threats (Rutjes et al., 2023; Callihan et al., 2021).

Key actions include: national guidance and dedicated funding for ISO 35001 implementation (International Organization for Standardization, 2019), particularly for public-sector laboratories that may lack resources for compliance; periodic external audits—conducted by accredited assessors—that examine both biosafety and biosecurity dimensions, including physical security, personnel reliability, and waste management; and integration of occupational-health measures (vaccination, personal protective equipment, mental-health support, and exposure-response protocols) into biorisk management programs (Blacksell et al., 2024).

The integration of occupational health is particularly important given the cumulative stress on laboratory staff. Preliminary survey evidence involving biosafety professionals reported burnout, anxiety, and moral distress during the pandemic, although the generalizability of these findings remains uncertain (Gillum, 2025b).

#### 3.1.4 Address cyberbiosecurity

Given growing digital dependence in laboratory operations, national programs should mandate cyberbiosecurity risk assessments for facilities handling high-consequence materials or DURC data. Crawford et al. recommend mapping cyber-physical assets, analyzing potential impacts of compromise, and embedding cybersecurity controls directly into existing biosafety and biosecurity protocols—rather than treating them as separate domains (Crawford et al., 2023).

National cybersecurity agencies should collaborate with health and research sectors to: issue baseline cyberbiosecurity standards tailored to different laboratory types and risk levels; share threat intelligence on cyber campaigns targeting biological research facilities or health infrastructure; and support coordinated incident response when cyber and biological risks intersect (Crawford et al., 2023).

Specific vulnerabilities that should be addressed include networked building management systems that control laboratory air pressure and containment; DNA synthesis ordering platforms that could be manipulated to bypass screening; laboratory information management systems (LIMS) that contain sensitive experimental data; and cloud-based genomic analysis pipelines where sequence data from high-consequence pathogens may be stored (Nuclear Threat Initiative, 2025).

#### 3.1.5 Mitigate insider threat

Effective insider-threat programs may combine background screening, role-based access controls, behavioral awareness training, confidential reporting mechanisms, and a positive security culture, without relying on any single measure (Murray et al., 2023; Gillum, 2025b).

Building on this evidence, the national program should: require clear, documented role-based access controls for biological materials, equipment, and sensitive data, with regular audits of access logs; establish confidential mechanisms—including anonymous reporting channels and designated ombudspersons—for staff to report concerning behavior without fear of reprisal; train managers and supervisors to identify and appropriately escalate documented security-relevant behaviors or policy violations using standardized criteria, due-process safeguards, and protections against discriminatory profiling; and provide supportive leadership and accessible mental-health services to reduce grievances, burnout, and financial stress among staff with critical access privileges.

The emphasis on culture is deliberate. Overly surveillant approaches may erode trust, increase stress, and discourage the reporting of concerns or errors. Evidence from healthcare and other high-reliability sectors suggests that a just culture, in which staff are encouraged to report concerns and errors without disproportionate punishment, may support earlier identification of safety problems and more open incident reporting (Murray et al., 2023).

#### 3.1.6 Promote responsible communication and open science safeguards

Hoffmann and Gillum both highlight tensions between open science and security: the same transparency that enables scientific progress and public accountability can also lower barriers for malicious actors (Hoffmann et al., 2023; Gillum, 2025a). National guidance should address this tension through practical, proportionate measures rather than blanket restrictions.

Specific recommendations include: encouraging pre-submission DURC review by institutions and journals for manuscripts that describe high-consequence methods or data; developing criteria—in consultation with publishers, funders, and security agencies—for redacting or delaying release of specific methodological details when the foreseeable harm outweighs the benefits of immediate disclosure; and promoting secure data-sharing platforms with tiered access controls for high-risk datasets, enabling *bona fide* researchers to access information while maintaining audit trails.

The publication of the full genome sequence of the 1918 influenza virus in 2005—and the subsequent reconstruction of the virus—remains a landmark case study in how open-access genomic data can generate dual-use concerns (Evans, 2020). A recent policy report has highlighted similar tensions in the context of AI-enabled protein design and sequence-generation tools, which may facilitate the development of proteins with beneficial or potentially harmful functions (Nuclear Threat Initiative, 2025).

### 3.2 Response components

#### 3.2.1 Enhance early detection and surveillance

Distinguishing among naturally occurring outbreaks, accidental laboratory releases, and deliberate biological events is difficult, particularly early in an investigation (Cummings et al., 2021). Building on existing surveillance infrastructure, the national program should integrate DURC-sensitive indicators into routine epidemiological monitoring. These indicators include unusual geographic or temporal clusters of disease, particularly near high-containment laboratories; unexplained illness among laboratory or research staff; unusual genomic features that, when interpreted together with epidemiological, laboratory, and intelligence evidence, may warrant further investigation; and intelligence indicators of hostile interest in biological materials or research facilities. Genomic data alone should not be treated as proof of an accidental laboratory release or deliberate biological event.

The program should also ensure rapid, secure sharing of genomic, epidemiological, and laboratory data between health, research, and security agencies—governed by clear protocols that specify what data can be shared, with whom, and under what conditions (World Health Organization, 2022).

Establishing protocols for joint epidemiological–forensic investigation when an accidental laboratory release or deliberate biological event is reasonably suspected is essential. These protocols should designate lead agencies, define evidence-handling procedures that meet both public-health and law-enforcement standards, and specify international notification obligations under the International Health Regulations (2005) and, where applicable, the UN Secretary-General’s Mechanism for Investigation of Alleged Use of Chemical and Biological Weapons.

#### 3.2.2 Develop DURC-specific emergency response plans

Generic pandemic plans may be insufficient for DURC-related events, which may involve enhanced pathogens, criminal or national-security dimensions, and intense public scrutiny. Building on WHO guidance and experience from COVID-19, response plans should include: clear triggers—based on epidemiology, situational intelligence, and laboratory findings—for activating a DURC-specific incident response; pre-designated arrangements for rapid, multi-disciplinary risk assessment with input from biosafety, biosecurity, legal, and ethics experts (World Health Organization, 2022); mechanisms to temporarily restrict or adjust research activities at implicated facilities while investigations proceed, without permanently damaging scientific capacity; and procedures for international notification and cooperation, recognizing that DURC-related events may cross borders rapidly and may engage obligations under the Biological Weapons Convention.

Exercising these plans through regular tabletop and functional simulations—involving all relevant sectors—is critical for identifying gaps before a real event occurs. Multiagency exercises—such as those simulating zoonotic disease outbreaks with bioterrorism dimensions—have revealed significant coordination failures that were not apparent from written plans alone (Jackson et al., 2006).

#### 3.2.3 Protect first responders and healthcare workers

The program must ensure that first responders and healthcare workers are not disproportionately exposed to risk without adequate institutional protection during DURC-related events. Building on guidance from biosafety and COVID-19 literature (Rutjes et al., 2023; Blacksell et al., 2024), occupational-health plans should guarantee timely access to appropriate personal protective equipment (PPE), prophylaxis, and vaccines where available; provide training that familiarizes responders with the clinical and epidemiological signs of unusual or high-consequence biological incidents—distinguishing them from common infectious diseases; and include psychosocial support services and fair compensation mechanisms to sustain workforce resilience during prolonged or repeated responses.

The principle of reciprocity is important here: workers who accept elevated risk on behalf of society are owed a corresponding duty of care from the institutions that deploy them. This principle is articulated in the WHO framework (World Health Organization, 2022).

#### 3.2.4 Risk communication and public engagement

Mistrust and misinformation can amplify the harm from DURC-related incidents, as demonstrated during COVID-19 (Rutjes et al., 2023). The WHO framework stresses the importance of transparent, values-based communication that acknowledges uncertainty while avoiding speculation (World Health Organization, 2022).

The national program should designate trusted health and scientific spokespeople in advance, rather than scrambling to identify communicators during a crisis; prepare pre-agreed communication templates for scenarios including laboratory accidents, suspected deliberate release, and false alarms; and engage communities and civil-society organizations proactively—before any incident occurs—to discuss DURC, the ethical trade-offs involved in research oversight, and the mechanisms through which decisions are made (World Health Organization, 2022; Dietz, 2013).

Proactive engagement builds the reservoir of trust that crisis communication depends upon. Communities that understand why certain research is regulated, and how oversight decisions are made, are less likely to interpret restrictions as arbitrary or conspiratorial (Dietz, 2013).

#### 3.2.5 Continuous learning and international collaboration

Biological risks evolve rapidly, and Morse describes biosafety and biosecurity as a “grand challenge” that demands continuous adaptation, research, and shared learning (Morse, 2025). A national program should establish mechanisms to systematically review DURC-related incidents, near-misses, and exercises—disseminating lessons learned and updating policies accordingly; participate actively in international networks on DURC governance, biosafety, biosecurity, and cyberbiosecurity, including the WHO’s piloting initiative for the Global Guidance Framework (World Health Organization, 2023); and contribute to refining global frameworks, recognizing that biological threats do not respect borders and that strong national systems collectively strengthen international security.

Peer-review mechanisms—where countries voluntarily submit their DURC governance arrangements to external review by international counterparts, building on established tools such as the WHO Joint External Evaluation (JEE) framework—have been proposed as a tool for building mutual confidence and identifying improvement opportunities (World Health Organization, 2022).

### 3.3 Implementation priorities

Not all eleven recommendations carry equal urgency. Table 1 organizes them into a three-tier implementation sequence, reflecting the logic that foundational governance and surveillance must precede resource-intensive interventions. Table 2 provides a detailed evidence-strength assessment for each individual recommendation.

## 4 Discussion

### 4.1 Summary of key findings

This review has argued that DURC sits at the intersection of scientific innovation and potentially high-consequence biological risk. The expansion of high-risk biological capabilities—driven by advances in synthetic biology, gene editing, and AI—coupled with the increased connectivity of laboratories and demonstrated vulnerabilities in health systems, has made DURC-related biological incidents an increasingly important policy concern (Ienca and Vayena, 2018; Cummings et al., 2021; Hoffmann et al., 2023).

The proposed eleven-component program addresses this challenge across the full risk-management cycle. Prevention components—governance, oversight, laboratory management, cyberbiosecurity, insider threat mitigation, and responsible communication—are intended to reduce the probability of a DURC-related event. Response components—surveillance, emergency planning, worker protection, risk communication, and continuous learning—are intended to limit the consequences should prevention fail. The framework gives particular emphasis to frontline-worker protection, occupational health, and institutionalized intersectoral coordination, which this review identifies as comparatively underdeveloped or inconsistently integrated across the instruments examined.

### 4.2 Implementation challenges

Implementing a comprehensive national DURC program faces several significant challenges. First, resource constraints are considerable. The technical, human, and financial investments required—from ISO 35001 implementation to cyberbiosecurity assessments—may be prohibitive for lower-resource settings. Phased implementation, starting with the highest-risk laboratories and research activities, can make the program more manageable. International support may be available through collaborative initiatives such as the Global Health Security Agenda (GHSA) and financing mechanisms such as the Pandemic Fund (Global Health Security Agenda, 2024; Pandemic Fund, 2024).

Second, political will is uneven. DURC governance competes for attention with more immediate health priorities and may be perceived as constraining scientific competitiveness. Building coalitions that include scientific leaders, industry representatives, and security officials—and articulating the economic case for prevention—can help sustain political commitment.

Third, the rapid pace of technological change means that any governance framework risks obsolescence. The recommendations emphasize regular review and adaptation, but the institutional mechanisms for maintaining currency—such as horizon-scanning units and technology assessment processes—require dedicated resourcing.

To support practical application, the three-tier implementation sequence in Table 1 can be further adapted according to national capacity. These capacity categories are illustrative rather than fixed and should be assessed across statutory authority, regulatory workforce, surveillance systems, laboratory infrastructure, financing, cybersecurity capacity, and interagency coordination, rather than by containment level alone. High-capacity settings—with comparatively mature legal and regulatory institutions, sustainable financing, established surveillance and laboratory systems, digital-security capability, and functioning interagency coordination mechanisms—may feasibly pursue the full three-tier framework. Medium-capacity settings—with partial regulatory coverage or uneven capacity across these domains—may prioritize Tier one governance and surveillance components and Tier two laboratory biorisk management and worker-protection measures, while introducing selected Tier three components as institutional capacity develops. Low-capacity settings—with limited capacity across several of these dimensions—may begin with a Tier one minimum package comprising a national governance framework, basic surveillance integration, and participation in international assessment or peer-review mechanisms such as the WHO Joint External Evaluation. Subsequent components may then be phased in as legal, institutional, technical, and financial capacity matures. BSL-3 or BSL-4 facilities may indicate one dimension of technical capacity, but their presence or absence should not determine the overall national capacity category.

Implementation also depends on workforce, incentive, and compliance arrangements. Governments would require trained DURC reviewers, laboratory biorisk auditors, cyberbiosecurity specialists, occupational-health personnel, and multidisciplinary incident investigators. Compliance incentives could be linked to research funding, laboratory accreditation, and authorization to conduct high-consequence work, while monitoring could combine institutional self-assessment with proportionate external audits. Because additional oversight may generate administrative burdens and resistance among researchers and institutions, implementation should include technical assistance, stakeholder consultation, transparent appeal mechanisms, and pilot evaluation before national scale-up. These operational arrangements remain largely consensus-based and require empirical validation.

Fourth, and equally important, DURC governance is not an unqualified good: tightening oversight carries its own risks that must be acknowledged and managed. The most prominent concern is the potential chilling effect on legitimate research. A master’s thesis examining Denmark’s 2008 Biosecurity Act estimated a 46% reduction in biotechnology patent output following its implementation, suggesting that poorly calibrated regulation can suppress innovation across the sector, not only in high-consequence research, though this finding has not been independently corroborated (Onkenhout, 2024). If compliance burdens are perceived as excessive, researchers may abandon valuable lines of inquiry, relocate to less-regulated jurisdictions, or shift to private-sector environments where oversight is thinner—outcomes that would paradoxically reduce, rather than enhance, collective biosecurity. Publication review mechanisms, while necessary for particularly sensitive findings, must be designed with sufficient precision to avoid creating a *de facto* prior-restraint regime that conflicts with norms of scientific openness (Hoffmann et al., 2023; Evans, 2020).

Equity considerations are similarly underappreciated. The framework described here assumes a degree of institutional infrastructure, regulatory capacity, and fiscal space that may be absent in many low- and middle-income countries. If international norms for DURC governance coalesce around high-income-country standards, countries with nascent life-science sectors could face implicit barriers to research participation, technology transfer, and scientific collaboration—effectively externalizing the governance burden onto those least equipped to bear it (Gillum, 2025b). A tiered implementation model, combined with technical cooperation through initiatives such as the GHSA and dedicated financing through mechanisms such as the Pandemic Fund, may partially mitigate this dynamic but cannot eliminate it (Global Health Security Agenda, 2024; Pandemic Fund, 2024).

Finally, insider-threat programs warrant particular scrutiny. Background checks, behavioral monitoring, and confidential reporting systems can, if implemented without robust procedural safeguards, create climates of suspicion, erode laboratory morale, and expose staff—particularly those from marginalized or foreign-national backgrounds—to discriminatory scrutiny. The “just culture” approach recommended in Section 3.1.5 is intended to counterbalance these tendencies, but its effectiveness depends on institutional leadership that is genuinely committed to fairness rather than performative compliance (Murray et al., 2023). Regular independent review of insider-threat program outcomes—including demographic impact assessments—should be embedded in the governance framework from the outset.

### 4.3 Governance debates and unsettled issues

Several governances debate relevant to this framework remain unsettled and warrant acknowledgment. First, the relationship between DURC and gain-of-function (GOF) research continues to generate disagreement. While DURC refers broadly to legitimate research that could be misapplied, GOF research specifically involves experiments that enhance pathogen properties such as transmissibility or virulence. Not all GOF research qualifies as DURC under existing definitions, yet the two categories overlap considerably in practice, and their conflation in policy discourse has at times muddied regulatory design. This framework adopts an inclusive scope but does not resolve the ongoing debate about whether GOF research with pandemic-potential pathogens warrants distinct governance treatment. Second, terminology itself is in flux. The United States formalized combined DURC and PEPP terminology in the 2024 DURC-PEPP Policy (United States Office of Science and Technology Policy, 2024), while Executive Order 14,292 subsequently emphasized “dangerous gain-of-function research” and directed revision or replacement of the 2024 policy (Executive Office of the President, 2025). These terms are not used consistently across jurisdictions: the WHO Global Guidance Framework, the Biological Weapons Convention, and many national systems continue to use broader DURC or biorisk concepts. This manuscript therefore uses DURC and PEPP where appropriate while acknowledging that international terminology and U.S. federal policy remain unsettled. Third, governance approaches differ substantially across countries. The United States has historically relied heavily on conditions attached to federally sponsored research (United States Office of Science and Technology Policy, 2024), whereas the European Union has emphasized export controls and dual-use item regulation (European Union, 2021). In many lower-resource settings, biosafety, laboratory capacity-building, and broader health-security preparedness may remain more immediate priorities than formal DURC-specific oversight (World Health Organization, 2022; World Health Organization, 2023; Oladimeji et al., 2019). These divergent paths reflect genuine differences in legal traditions, research funding models, and institutional capacity. The framework proposed here is not intended to replace these national approaches but to offer a common organizing structure adaptable to different governance contexts.

### 4.4 Limitations of this review

This review has several limitations. First, it draws primarily on English-language literature and frameworks from high-income countries, particularly the United States and Europe. DURC governance challenges in low- and middle-income countries—where research capacity is growing but regulatory infrastructure may be less developed—deserve dedicated analysis that this review cannot provide (Oladimeji et al., 2019). Second, although domestic private-sector laboratories are included as stakeholders in the proposed framework, the review does not examine in detail the governance of multinational pharmaceutical and biotechnology research spanning multiple regulatory jurisdictions. Third, empirical evidence on the effectiveness of specific DURC governance measures is limited. Most recommendations rest on expert consensus, case studies, and analogies from related domains (such as nuclear safety and information security) rather than controlled evaluations—a gap that the research community should address. Fourth, several arguments rely on grey literature, including a doctoral dissertation, a master’s thesis, and policy-institute reports. These sources were retained because they provide policy, survey, or implementation evidence not readily available in peer-reviewed publications. Nevertheless, findings supported primarily by these sources should be regarded as preliminary and require independent corroboration.

Fifth, the framework has not been validated through stakeholder consultation, Delphi consensus methodology, or formal review by policymakers or biosafety professionals. The recommendations represent the author’s synthesis of available literature and policy instruments, and their practical feasibility, acceptability, and effectiveness have not been empirically tested.

Sixth, incomplete records from the original coursework-based search prevent reconstruction of the numbers initially identified, screened, and excluded; this limits reproducibility and may have introduced selection bias.

### 4.5 Implications for policy and practice

Despite these limitations, the convergence of international guidance, national policy developments, and academic analysis provides a practical but provisional basis for policy development, adaptation, and pilot evaluation. Countries that adapt and test the components described in this review may strengthen preparedness for DURC-related biological incidents and broader biological threats.

The recommendations are not prescriptive in detail and should be adapted to national legal systems, research ecosystems, institutional capacity, and risk profiles. Together, they form a proposed integrated national DURC risk-management architecture that requires context-specific adaptation and evaluation before large-scale implementation.

### 4.6 Future research directions

Several research priorities emerge from this review. First, comparative case studies of national DURC governance implementation—including barriers, adaptations, and outcomes—would strengthen the evidence base for policy design. Second, empirical research on the effectiveness of specific oversight mechanisms (e.g., institutional DURC review committees, ISO 35001 implementation, cyberbiosecurity assessments) is needed to move beyond expert consensus. Third, the governance implications of AI-enabled biological design tools deserve dedicated attention, as current frameworks were developed before the recent acceleration in this field. Fourth, research on public perceptions of DURC governance—and on methods for meaningful public engagement in oversight decisions—would support the legitimacy and sustainability of regulatory systems. Fifth, economic modeling of the costs and benefits of DURC prevention could strengthen the investment case for resource-constrained settings.
